# A Novel, Inexpensive In-House Immunochromatographic Strip Test for Cryptococcosis Based on the Cryptococcal Glucuronoxylomannan Specific Monoclonal Antibody 18B7

**DOI:** 10.3390/diagnostics11050758

**Published:** 2021-04-23

**Authors:** Pakornswit Sathongdejwisit, Kritsada Pruksaphon, Akarin Intaramat, Pisinee Aiumurai, Nitat Sookrung, Kavi Ratanabanangkoon, Joshua D. Nosanchuk, Sirida Youngchim

**Affiliations:** 1 Department of Microbiology, Faculty of Medicine, Chiang Mai University, Chiang Mai 50200, Thailand; pakornswit@gmail.com (P.S.); kpruksaphon@gmail.com (K.P.); 2 Translational Research Unit and Laboratory of Immunology, Chulabhorn Research Institute, Bangkok 10210, Thailand; akarin@cri.or.th; 3 Biomedical Research Incubation Unit, Department of Research, Faculty of Medicine Siriraj Hospital, Mahidol University, Bangkok 10700, Thailand; hedhom_@hotmail.com (P.A.); nitat.soo@gmail.com (N.S.); 4 Department of Microbiology, Faculty of Science, Mahidol University, Bangkok 10400, Thailand; kavi.rtn@mahidol.ac.th; 5 Departments of Medicine (Division of Infectious Diseases) and Microbiology and Immunology, Albert Einstein College of Medicine, Bronx, NY 10461, USA; josh.nosanchuk@einsteinmed.org

**Keywords:** *Cryptococcus neoformans*, cryptococcosis, immunodiagnosis, immunochromatography, monoclonal antibody 18B7, glucuronoxylomannan

## Abstract

The aim of this study was to develop a novel lateral flow immunochromatoghaphic strip test (ICT) for detecting cryptococcal polysaccharide capsular antigens using only a single specific monoclonal antibody, mAb 18B7. The mAb 18B7 is a well characterized antibody that specifically binds repeating epitopes displayed on the cryptococcal polysaccharide glucuronoxylomannan (GXM). We validated the immunoreactivities of mAb 18B7 against capsular antigens of different cryptococcal serotypes. The mAb 18B7 ICT was constructed as a sandwich ICT strip and the antibody serving in the mobile phase (colloidal gold conjugated mAb 18B7) to bind one of the GXM epitopes while the stationary phase antibody (immobilized mAb18B7 on test line) binding to other remaining unoccupied epitopes to generate a positive visual readout. The lower limit of detection of capsular antigens for each of the *Cryptococcus* serotypes tested was 0.63 ng/mL. No cross-reaction was found against a panel of antigens isolated from cultures of other pathogenic fungal, except the crude antigen of *Trichosporon* sp. with the lower limit of detection of 500 ng/mL (~800 times higher than that for cryptococcal GXM). The performance of the mAb 18B7 ICT strip was studied using cerebrospinal fluid (CSF) and serum and compared to commercial diagnostic kits (latex agglutination CALAS and CrAg IMMY). The sensitivity, specificity and accuracy of the mAb18B7 ICT with CSF from patients with confirmed cryptococcal meningitis were 92.86%, 100% and 96.23%, respectively. No false positives were observed with samples from non-cryptococcosis patients. With serum samples, the mAb 18B7 ICT gave a sensitivity, specificity and accuracy of 96.15%, 97.78% and 96.91%, respectively. Our results show that the mAb 18B7 based ICT was reliable, reproducible, and cost-effective as a point-of-care immunodiagnostic test for cryptococcosis. The mAb 18B7 ICT may be particularly useful in countries where commercial kits are not available or affordable.

## 1. Introduction

*Cryptococcus neoformans* is an opportunistic pathogenic fungus that causes cryptococcosis in immunocompromised patients, especially in advanced HIV/AIDS [1]. The prevalence of cryptococcal infection is highest in low-income and middle-income countries due to the burden of HIV infection [2]. Although the lung is the initial site of infection, *C*. *neoformans* frequently disseminates and is notorious for causing cryptococcal meningitis [3]. At the peak of the HIV pandemic, cryptococcosis caused over 600,000 deaths annually [4]. Increased treatment efforts, particularly following the World Health Organization (WHO) recommendations for antiretroviral therapy (ART) in HIV infected patients, have improved the health of afflicted patients and reduced the incidence of cryptococcosis, but cryptococcosis continues to kill ~180,000 people every year [2].

There are several laboratory methods for the diagnosis of cryptococcosis and newer approaches have markedly reduced the complexity and time for achieving results. The definitive (confirmatory) methods are direct examination of the fungus in body fluids with India ink preparation, histopathological staining of infected tissues and microbiological cultures, with culture being the “gold standard” For the conventional definitive method, India ink preparation and fungal culture demonstrated a high degree of diagnostic specificity, but the diagnostic sensitivity is insufficient. The sensitivity of India ink in cerebrospinal fluid (CSF) is approximately 72% compared to that of the culture method [5], and the sensitivity of fungal culture is approximately 50–70% for blood culture [6]. Therefore, immunochemical based diagnosis such as enzyme immunoassay, reverse passive latex agglutination and lateral flow immunochromatography are widely used instead due to their advantages with accuracy, simplicity, and quickness [7].

Early work revealed that cryptococcal capsular polysaccharide antigens were detected more often than cryptococcal specific antibodies in patient serum [8]. Therefore, various immunodiagnostics based on cryptococcal capsular polysaccharide antigen detection have been created and used extensively [9,10,11]. Recently, the WHO has recommended the use of Cryptococcal Antigen Latex Agglutination System or CALAS (Meridian Bioscience, Cincinnati, OH, USA) and Cryptococcal Antigen Lateral Flow Assay or CrAg LFA (IMMY, Norman, OK, USA) as the preferred reference diagnostics for cryptococcosis in patients with HIV [12]. However, these assays are very costly and are not affordable in many developing countries, especially in Southeast Asia including Thailand. With limited resources, the development of an in-house lateral flow format strip is urgently needed. Such tests, as recommended by the WHO for use in low-or middle-income countries, should be sensitive, specific, user-friendly, rapid and robust, equipment-free, affordable and deliverable to end-users [13].

The cryptococcal capsule is certainly the most prominent virulence factor in *C. neoformans/C. gattii* [3]. The main components of cryptococcal capsular polysaccharide antigens are glucuronoxylomannan (GXM) and glucuronoxylomannogalactan (GXMGal). Ninety percent of the capsule mass is composed of GXM and is generally referred to as cryptococcal antigen or CrAg [14,15,16]. For this reason, GXM is the major antigenic determinant of cryptococcal capsular antigens, and it has been the main antigenic target to generate monoclonal antibodies (mAbs) or polyclonal antibodies for use in antigen detection. One such well characterized mAb is mAb 18B7 (mouse immunoglobulin G1; IgG1κ), which was produced from a BALB/c mouse immunized with purified GXM conjugated tetanus toxoid [17,18]. This mAb has been used in a phase 1 study in patients with cryptococcosis [19].

The objective of the current study was to develop a mAb18B7 sandwich-based lateral flow immunochromatoghaphic strip test for screening cryptococcal antigen in clinical serum and CSF specimens. The mAb 18B7 conjugated to 40 nm of gold colloid particle was used as a specific signal reporter. In addition, the mAb 18B7 was functionally used again by immobilization at the test line zone to capture the GXM capsular antigen. The diagnostic performances of the novel sandwich ICT were assessed in comparison to current reference detection strips (latex agglutination, CALAS and CrAg LFA, IMMY) using clinical samples from patients with or without cryptococcosis.

## 2. Materials and Methods

### 2.1. Preparation of C. neoformans Capsular Antigens and Other Pathogenic Fungal Antigen

#### 2.1.1. Fungal Isolates

*C. neoformans* H99 (serotype A), *C. neoformans* CBS 8710 (serotype A), *C. deuterogattii* CBS 6956 (serotype B), *C. gatii* CBS 14407 (serotype B) and *C. deneoformans* CBS 10513 (serotype D) were cultured on potato dextrose agar (PDA: Difco) and incubated at 25 °C for 3 days. Then, yeast cells were harvested by suspension in phosphate buffer saline (PBS). Thereafter, 2.5 × 10^6^ cells/mL of *C*. *neoformans* were inoculated in minimal medium broth (15 mM Glucose, 10 mM MgSO_4_, 29.4 mM KH_2_PO_4_, 13 mM Glycine and 3 μM Thiamine) and incubated at 30 °C with 150 rpm shaking for 3 days. The culture broth was then centrifuged at 4500 rpm for 30 min to separate the cell pellet. The supernatant was collected and centrifuged at 10,000 rpm, 30 min. The supernatant was collected and stored at 4 °C. Other pathogenic fungal species were obtained and cultivated according to the directions from the American Type Culture Collection (ATCC) or from Department of Medical Services, Ministry of Public Health, Bangkok, Thailand. The fungal strains used in this work are summarized in Table 1.

#### 2.1.2. Purification of Capsular Antigens of *C. neoformans*

The capsular antigens were purified and concentrated by ultrafiltration using Vivacell 250 and ultrafiltration membrane with 100 kDa molecular weight cut-off as described by Nimrichter et al. (2007) [20]. Gelatinous like capsular antigens on the membrane were transferred to centrifuge tubes and kept at 4 °C. The total concentration of *C. neoformans* capsular antigens was quantified by phenol-sulfuric acid method (Total carbohydrate assay kit; Abcam) using D-glucose as the standard [16]. 

#### 2.1.3. Crude Fungal Cytoplasmic Protein Antigen Extraction

Extraction of crude cytoplasmic antigen was carried out as described previously [21,22]. The cytoplasmic antigens of yeast cells (CYA) or mycelial cells (CMA) of other human pathogenic fungi including *C. neoformans* strain H99 (serotype A) were also prepared following this standardized procedure. The fungal protein concentrations of the individual preparations were determined by the Coomassie Brilliant Blue G-250 binding method (Bio-Rad Labs, Hercules, CA, USA) using bovine serum albumin (BSA) as the standard [21,23]. 

### 2.2. Production and Purification of Monoclonal Antibody 18B7 (mAb18B7)

The murine derived hybridoma cell line clone 18B7, IgG 1 isotype, (gift from Arturo Casadevall, M.D., Ph.D., Johns Hopkins University, Baltimore, MD, USA) was cultured in hybridoma serum-free medium (SFM; Gibco, Waltham, MA, USA) and then purified by using HiTrap column protein G affinity chromatography (GE Healthcare, Chicago, IL, USA) as described by Pruksaphon et al. (2018) [23]. The purity of the IgG fraction was evaluated with 10% SDS-PAGE. The concentrations of purified mAb 18B7 were determined by molar extinction coefficient at 280 nm of purified IgG (1.36 for a solution of 1 mg/mL) [24]. The immunoreactivity of mAb 18B7 was confirmed using indirect ELISA as described below. 

### 2.3. Determination of the Immunoreactivity of mAb18B7 by Enzyme-Linked Immunosorbent Assay (*ELISA*)

#### 2.3.1. Determination of the Cross-Reactivity of mAb 18B7 with Other Fungal Pathogens by Indirect ELISA

To determine the cross-reactivity of mAb 18B7 with other pathogenic fungal antigens, 50 μL of GXM of strain H99 and crude cytoplasmic protein antigens of other pathogenic fungi were coated into 96-well polystyrene plate (Nunc A/S, Kamstrup, Denmark). The optimized concentrations of capsular antigen and other pathogenic fungi were 10 µg/mL and the incubation was at 4 °C overnight. Excess unbound antigen was removed by washing with 0.05% Tween 20 in phosphate buffer saline (PBST). BSA (1.5% *w*/*v*) in PBST was added and the plate was incubated at 37 °C for 1 h. After that, 50 µL of mAb 18B7 (20 ng/mL) was added and incubated at 37 °C for 1 h. The plates were washed again, 50 μL of HRP-conjugated goat anti-mouse IgG antibody (Jackson, West Grove, PA, USA) in 1:10,000 was added and incubated at 37 °C for 1 h. After washing, H_2_O_2_/TMB substrate (BioFX Laboratories, SurModics IVD, Eden Prairie, MN, USA) was added and the enzymatic reaction was allowed to take place for 15 min at room temperature in the dark. The reaction was stopped by adding 2N H_2_SO_4_. The OD450 was measured against a reference at OD570 on an ELISA plate reader (Shimadzu model UV-2401PC, Kyoto, Japan). The test was performed in triplicates.

#### 2.3.2. Determination of the Immunoreactivity by Inhibition ELISA 

The immunoreactivity of mAb 18B7 against capsular antigens of various strains of *Cryptococcus* sp. was determined by an inhibition ELISA. The capsular antigen of strain H99 at concentration of 2 µg/mL in 2× PBS (50 µL) was immobilized on 96 well plates at room temperature overnight. Thereafter, the plates were washed three times with PBST and then blocked with 1.5% (*w*/*v*) BSA in PBST at 37 °C for 1 h. 

Each capsular antigen of the Cryptococcus serotypes was diluted in PBST to give serial concentrations of 0.2, 0.4, 0.78, 1.56, 3.125, and 6.25 ng/mL in microcentrifuge tubes. After that, each concentration (100 µL) was mixed with 100 µL of 40 ng/mL of mAb 18B7 (giving the final concentration 0.1, 0.2, 0.4, 0.78, 1.56, and 3.125 ng/mL of capsular antigen, respectively) and then incubated at 37 °C for 1.5 h. Each of these mixtures (50 µL) was added to the H99 capsular antigen coated plates followed by incubation at 37 °C for 1 h. The remaining procedures of the indirect ELISA were followed as described above. 

### 2.4. Development of mAb 18B7 Based Lateral Flow Immunochromatographic Test (mAb 18B7 ICT Strip)

#### 2.4.1. Principle of Assay Format

The mAb 18B7 based sandwich ICT strip was developed to determine cryptococcal capsular antigen in serum and cerebrospinal fluid (CSF) samples. The mAb 18B7-colloidal gold (CG) conjugate was used to produce the assay signal. The mAb 18B7 was immobilized on the test line and goat anti-mouse IgG antibody was immobilized on the control line of the micro-porous analytical nitrocellulose membrane. The ICT starts with an absorbing pad at the lower end that enables the specimen fluid to move along the strip via capillary tension to rehydrate the mAb 18B7-CG conjugate, which then flows along the sample application pad.

**Figure 1:**
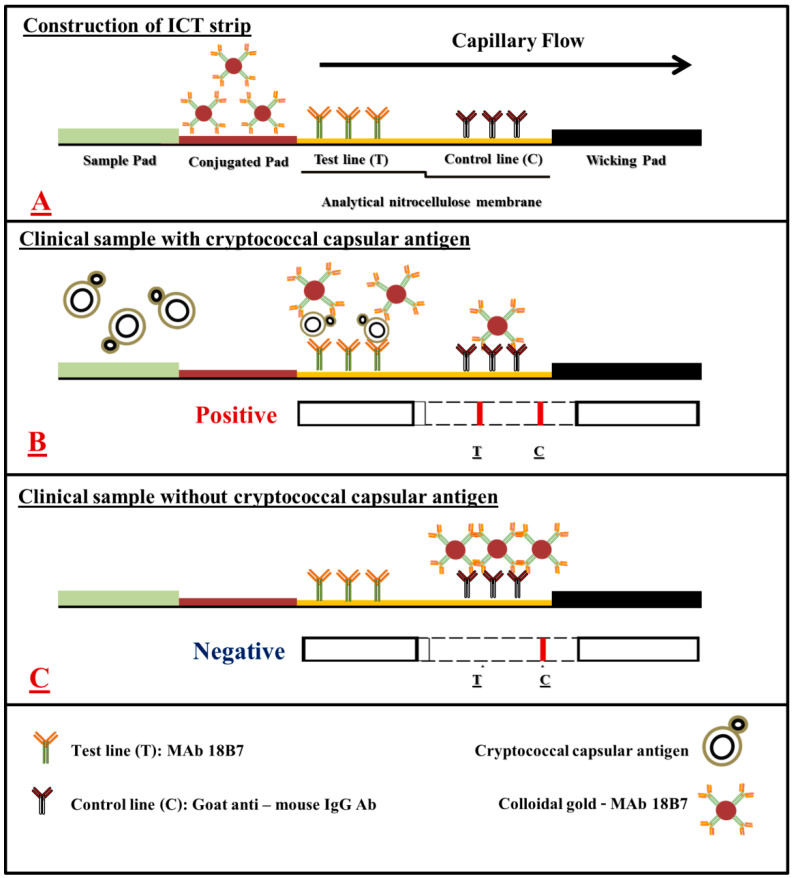
A schematic of mAb 18B7 sandwich ICT strip for the rapid detection of cryptococcal capsular antigen from clinical samples. (**A**), the schematic presentation of the positions where colloidal gold conjugated mAb 18B7, mAb 18B7 and goat anti-mouse IgG antibodies are immobilized in an analytical nitrocellulose membrane. (**B**), the reactions that occur on the mAb 18B7 sandwich ICT strip in the presence of cryptococcal capsular antigen and (**C**), in the absence of cryptococcal capsular antigens. The red–purple color of colloidal gold conjugate appears at the test line and/or control line, depending on the presence or absence of cryptococcal capsular antigen in the clinical sample.

**Figure 2:**
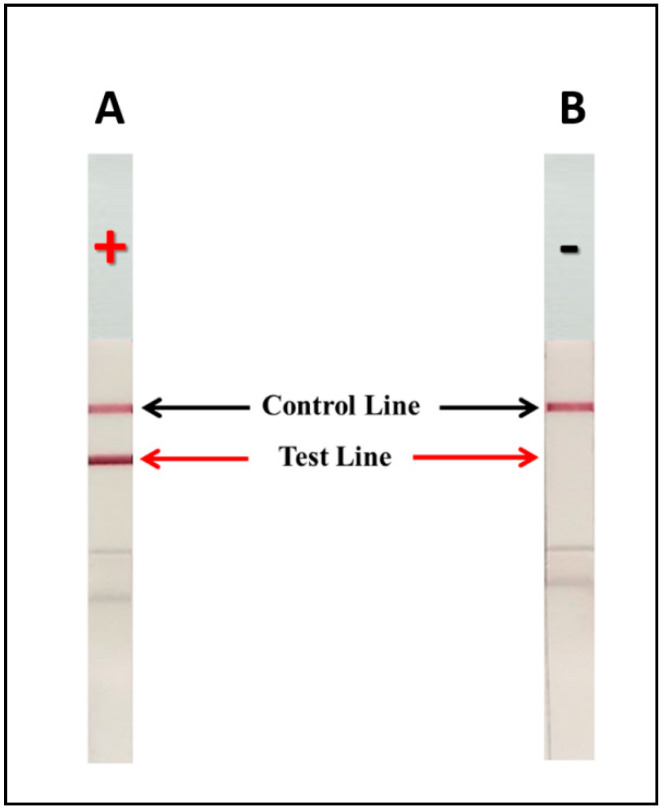
Depiction of the mAb 18B7 sandwich ICT strip with or without detection of cryptococcal capsular antigen. (**A**): a positive result (+) showing intense signals at both the test and at the control lines. (**B**): a negative result (−) showing signal only at the control line.

When cryptococcal capsular antigen is present in the specimen, the antigen will bind to the mAb 18B7-CG conjugate and will be captured by the free mAb 18B7 immobilized at the test line (T). This establishes a colored test line and represents a positive result. If the cryptococcal capsular antigen is absent or below the cut-off point (lower than the limit of detection), there is no visible color at the test line (T), and this represents a negative result. As an internal control feature, immobilized goat anti-mouse IgG antibody reacts with the mAb 18B7-CG conjugate to form a visible control line (C), indicating that the assay has been performed properly. The entire process takes about 30 min (Figure 1 and Figure 2). 

#### 2.4.2. Preparation of Colloidal Gold Nanoparticle—mAb 18B7 Signal Reporter

The mAb 18B7-colloidal gold (mAb 18B7-CG conjugate) is the signal reporter used for the detection of cryptococcal capsular antigen in clinical samples. The mAb 18B7 was conjugated to CG particle (40 nm in diameter) by isoelectric point dependent passive adsorption [25]. CG suspension (40 mL, Serve Science, Bangkok, Thailand) was adjusted to pH 8.0–8.5 with 0.1 M K_2_CO_3_. Purified mAb 18B7 in deionized water (final concentration 5 µg/mL) was added to the CG suspension and continuously mixed for 60 min at room temperature. The unoccupied surface of the CG particle was blocked with of 1% (*w*/*v*) BSA dissolved in 0.15 M of PBS pH 7.2 for 30 min. After that, the mAb 18B7-CG conjugate was added with 10% (*w*/*v*) sucrose (Sigma) and 5% (*w*/*v*) trehalose (Sigma). To prepare the conjugate releasing pads (CRP), a piece of glass fiber filter (Ahlstrom 8964; Ahlstrom, Finland) was used and pre-treated with pre-treat solution [1% (*w*/*v*) BSA, 1% (*w*/*v*) PVP-40, 0.25% (*v*/*v*) Triton X-100 dissolved in 2 mM Na_2_B_7_O_4_ pH 8.0], the treated glass fibers were dried at 37 °C overnight. Subsequently, the mAb 18B7-CG conjugate suspension was filled to the treated glass fibers by the XYZ3210 dispenser platform (BioDot) with rate transfer of 1 μL/cm. The sprayed conjugation pad was incubated and dried at 37 °C overnight before being assembled into the ICT strips.

#### 2.4.3. Establishment of Test Line and Control Line onto Analytical Nitrocellulose Membrane

To prepare an analytical nitrocellulose membrane, each antibody was immobilized onto a nitrocellulose membrane (AE99; Whatman Schleicher & Schuell, Dassel, Germany) using XYZ3210 dispense platform (BioDot) at rate of 1 µL/cm. Purified mAb 18B7 (0.5 µg/mL) and goat anti-mouse IgG antibody (1 mg/mL; Jackson, West Grove, PA, USA) were immobilized at the test line and the control line, respectively. The immobilized membranes were incubated at 37 °C for 60 min. After incubation, the immobilized membranes were blocked with membrane blocking buffer [1% (*w*/*v*) BSA, 1% (*v*/*v*) PVP–40, 0.25% (*v*/*v*) Triton X-100, dissolved in 2 mM Na_2_B_7_O_4_ pH 8.0] to prevent nonspecific binding and minimized background signals. The blocked membrane was incubated at 37 °C for 90 min and was ready for assemble into ICT strips.

#### 2.4.4. Preparation of the Sample Application Pad

To prepare the sample application pad, a single layer matrix membrane (Fushion 5; Whatman Schleicher & Schuell, Germany) was used. The membrane was pre-treated with 1 M Tris pH 8.0 at 37 °C overnight before being assembled into the ICT strip.

#### 2.4.5. Construction and Material Composition of ICT Strip

There are four major components of ICT strip which includes:

- Analytical membrane (Cellulose nitrate membrane AE99; Schleicher & Schuell; size 0.4 × 2.5 cm^2^)
- The mAb18B7–CG absorbed conjugation releasing pad (Ahlstrom 8964; Glass fiber, Ahlstrom, Finland; size 0.3 × 1.0 cm^2^)
- Sample application pad (Fushion 5; Whatman Schleicher & Schuell, Germany; size 0.4 × 1.75 cm^2^)
- Adsorbing (wicking) pad (Whatman; Schleicher & Schuell, Germany size 0.4 × 1.8 cm^2^).

**Figure 3:**
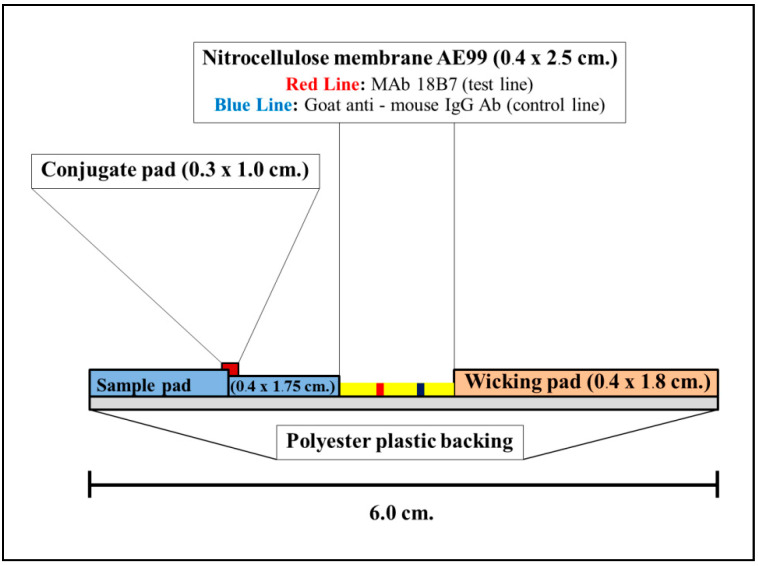
A side view of the mAb 18B7 sandwich ICT strip.

The above components were assembled manually and placed on laminating plastic backing (GL-187^®^; Lohmann; size 0.4 × 6.0 cm^2^). The laminate was cut into 0.4 cm by BioDot CM 4000 R guillotine cutter. The construction of strip is shown in Figure 3. 

### 2.5. Determination of the Lower Limit of Detection (LOD), Cross-Reactivity and Pan Serotype Specific of the mAb 18B7 ICT Strip

To evaluate the LOD of the mAb 18B7 ICT strip, the ICT strip was performed with capsular antigens of *C. neoformans* serotype A (H99). The capsular antigens were serially diluted from 1000 to 0.04 ng/mL in running buffer (150 mM NaCl, 10 mM PBS) before adding 100 µL to each well of 96 well plates, which allowed for a much more efficient assessment compared to using numerous strip tests. The lowest concentration of capsular antigen that showed positive result was considered as the LOD. 

To determine the cross-reactivity of the mAb 18B7 ICT strip, the ICT strip was tested with other crude fungal antigens listed in Table 1. The crude fungal antigens were dissolved in running buffer and used at 10 μg/mL. The capsular antigens of *C. neoformans* serotype A (H99) at 100 ng/mL was used as a positive control.

To study the pan-reactivity of the mAb 18B7 ICT strip against capsular antigen of various strains of *Cryptococcus* sp. The concentrations of capsular antigens were diluted in running buffer and tested at capsular antigen concentrations from 1000 to 0.04 ng/mL. The LOD of each serotype capsular antigens was determined. 

### 2.6. Evaluation of the Diagnostic Kit 

#### 2.6.1. Clinical Samples

The diagnostic performances of the mAb 18B7 ICT strip were evaluated with clinical samples that included serum and CSF. The confirmed cryptococcosis clinical samples were collected between 2017–2019 at the Clinical Microbiology Unit, Chiang Mai University Hospital, Chiang Mai, Thailand. The clinical samples were obtained at the time of diagnosis and stored at −20 °C and thawed immediately prior to investigation. For the non-cryptococcosis control group, serum samples from patients infected with other pathogenic microorganisms (fungus, virus, bacteria and parasites) or other diseases as well as normal healthy individuals were selected from our stored clinical samples collection and tested (Table 2). 

#### 2.6.2. Validation of Diagnostic Performance of the mAb 18B7 ICT Strip and Statistical Analysis 

We determined the diagnostic performance of the assay using 2 × 2 tables comparing mAb 18B7 ICT vs. standard commercial lateral flow assay (CrAg LFA, IMMY). The comparison was made on the number of true-positive results (TP), the true-negative results (TN), the false-positive results (FP), and the false-negative results (FN). The McNemar’s test for a case-control study was used to calculate the diagnostic sensitivity, specificity, accuracy and predictive values with their 95% confidence intervals (95% C.I.). Moreover, the consistencies between different tests were determined by Cohen’s kappa coefficient of agreement using GraphPad (QuickCalcs Program, San Diego, CA, USA), which was interpreted according to the kappa statistic values of >0.81, 0.41 to 0.80, or ≤0.40 indicating almost perfect agreement, moderate to substantial agreement, and slight to fair or poor agreement, respectively [26].

## 3. Results

### 3.1. The Immunoreactivity and Cross-Reactivity of mAb 18B7 against Cryptococcal Capsular Antigen and Other Fungal Pathogenic Antigens by Indirect ELISA 

**Figure 4:**
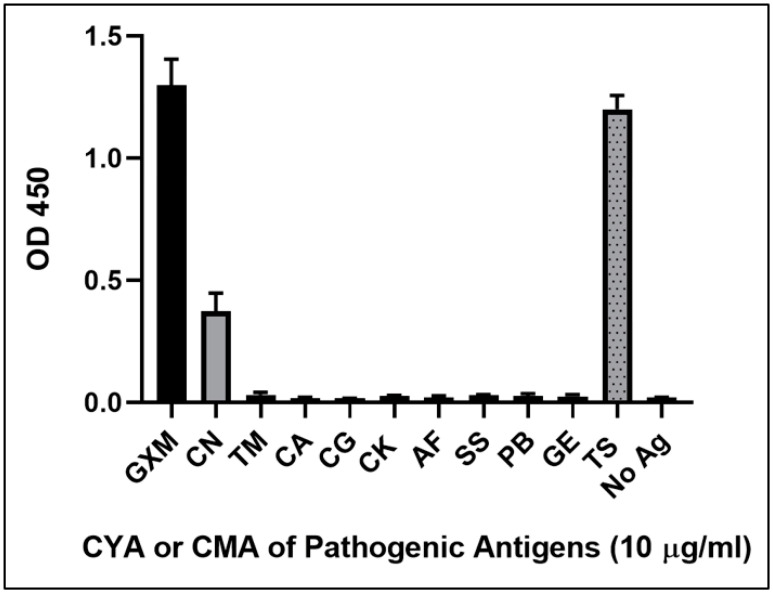
The various pathogenic fungal antigen and GXM capsular polysaccharide antigens were investigated for specificity by indirect ELISA. The absorbance measurement was observed at OD_450 nm_ with error bar represented as mean OD ± SD of triplicated determination. The concentration of GXM was 10 ng/mL. The concentrations of all pathogenic fungal antigens were 10 μg/mL. The cytoplasmic yeast antigens (CYA) of pathogenic fungi were indicated *C. neoformans* strain H99, serotype A (CN), *T. marneffei* (TM), *C. albicans* (CA), *C. glabrata* (CG), *C. krusei* (CK), *S. schenckii* (SS), and *Trichosporon* sp. (TS). The cytoplasmic mycelial antigens (CMA) were *A. fumigatus* (AF), *P. boydii* (PB), and *Geotrichum* sp. (GE).

The purified mAb 18B7 was investigated for immunoreactivity with GXM cryptococcal capsular antigen from strain H99 (serotype A). The optimized working concentration of mAb 18B7 was at 20 ng/mL. The CYA or CMA of other fungal pathogens was also assessed for binding with mAb 18B7 by indirect ELISA. The results clearly showed that the mAb 18B7 was able to efficiently react only with purified GXM capsular antigen or crude CYA of *C. neoformans* strain H99. However, crude CYA from *Trichosporon* sp. was also shown to cross-react with mAb 18B7 (Figure 4). 

### 3.2. The Immunoreactivity of mAb 18B7 against Other Serotypes of Cryptococcal Capsular Antigen as Studied by Inhibition ELISA 

**Figure 5:**
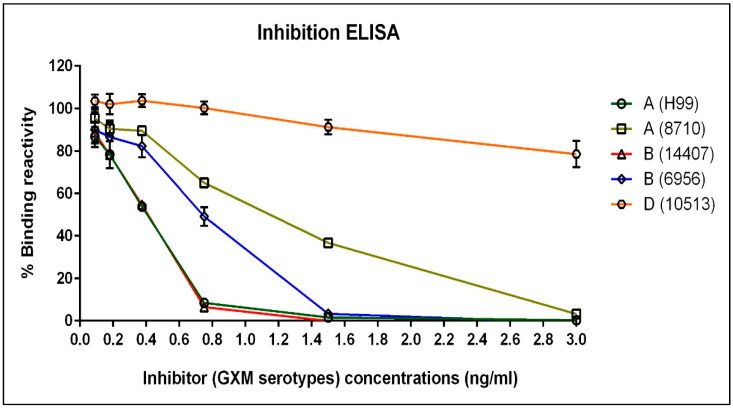
The immunoreactivity of mAb 18B7 with GXM derived from different serotypes of *Cryptococcus* sp. was investigated by an inhibition ELISA. The absorbance measurements were observed at OD_450 nm_ and were used to calculate the percentages of binding reactivities. The mAb 18B7 at 20 ng/mL was used in the inhibition ELISA. The error bar represented as mean OD ± SD of triplicated determination. The graph revealed the reactivities of GXM serotypes from high to low reactivity as follow; serotype A (H99) = B (14407) > B (6956) > A (8710) > D (10513), respectively. The A, B and D indicated the cryptococcal serotype and the name of each strain were indicated in the parenthesis.

The purified mAb 18B7 was optimized for immunoreactivity with purified capsular antigen from strain H99 (serotype A), as mentioned above. The purified capsular antigen of other cryptococcal strains was examined for the GXM serotypes reactivity with mAb 18B7 by inhibition ELISA. The data showed decreasing binding reactivity when the concentrations of antigenic inhibitors were increased. Strain H99 (serotype A) and strain 14407 (serotype B) were shown to strongly decrease the binding reactivity, which were different from other strains by at least 0.18 ng/mL of other serotype inhibitors. In addition, both strains, H99 and 14407, showed similar inhibition suggesting similar GXM serotype reactivity. However, when compared with strain H99, the other GXM serotype reactivity of strain 6956 (serotype B), strain 8710 (serotype A) and strain 10513 (serotype D) showed decreasing inhibition, respectively. Therefore, the results revealed the relative reactivities of GXM serotypes from high to low as follow; serotype A (H99) = B (14407) > B (6956) > A (8710) > D (10513) (Figure 5). 

### 3.3. Development of the mAb 18B7 ICT Strip for the Detection of Cryptococcal Capsular Antigen

A sandwich ICT strip based on mAb 18B7 for the detection of cryptococcal capsular antigen was developed using mAb 18B7 directly immobilized onto the analytical nitrocellulose membrane, while the mAb 18B7-CG conjugate served as the signal reporter. The specific reaction of cryptococcal capsular antigen and mAb 18B7-CG conjugate resulted in the formation of a visible red–purple immune complex on the test line of the nitrocellulose membrane. The excess mAb 18B7-CG conjugate moved further to the control line, which served to verify the validity of the assay (Figure 2). The process was completed within 30 min. The results were separately determined and recorded by three experienced laboratory technicians.

#### 3.3.1. Limit of Detection (LOD) of mAb 18B7 ICT Strip

**Figure 6:**
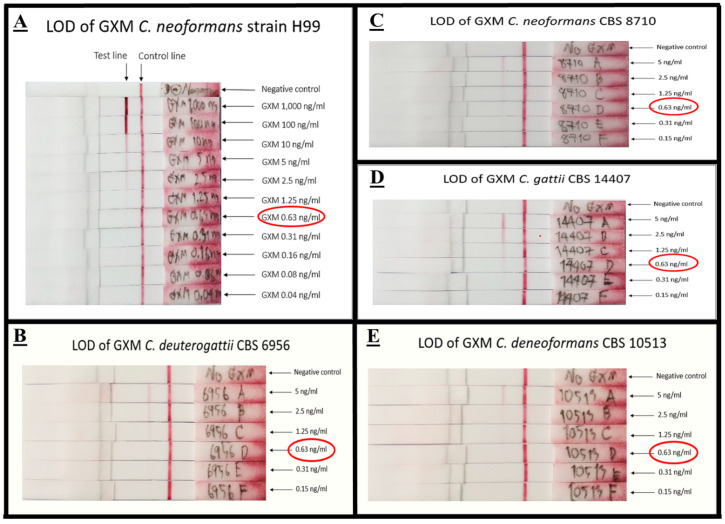
The LOD of mAb 18B7 sandwich ICT strip. The mAb 18B7 sandwich ICT strip was tested with GXM capsular antigen of serotype A, strain H99 (**A**), serotype B, strain CBS 6956 (**B**), serotype A, strain CBS 8710 (**C**) serotype B, strain CBS 14407 (**D**), and serotype D strain CBS 10513 (**E**). The ICT was measured by naked eye visual observation with three laboratory persons. The LOD of the mAb 18B7 sandwich ICT strip was 0.63 ng/mL for all cryptococcal serotypes. The red circle indicates the LOD was recorded.

Using an antigen spiking method, the LOD for the mAb 18B7 ICT strip against GXM of cryptococcal capsular antigen from strain H99 was determined by naked eye observation. The LOD of mAb 18B7 ICT strip with GXM cryptococcal capsular antigen of strain H99 was 0.63 ng/mL. In addition, LODs of mAb 18B7 ICT strip against GXM of other serotypes including serotype A, B, and D were 0.63 ng/mL. The LOD of each serotype is shown in Figure 6.

#### 3.3.2. Cross-Reactivity of mAb 18B7 ICT Strip

**Figure 7:**
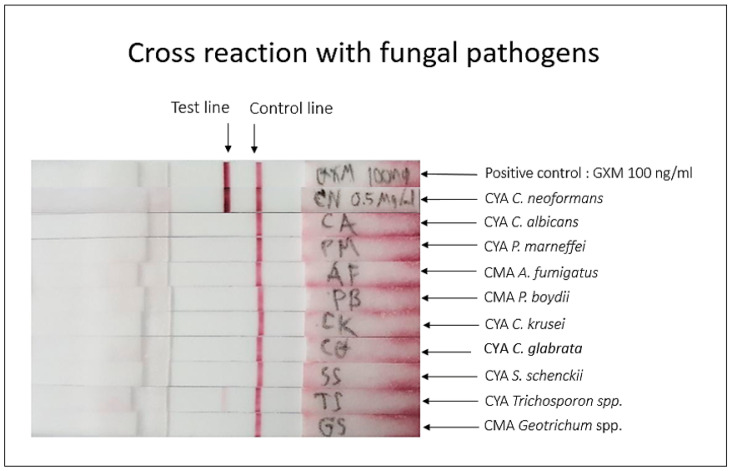
Cross-reactivity of mAb 18B7 sandwich ICT strip with fungal antigens from different pathogenic species. The cytoplasmic antigens of various pathogenic fungi were investigated the cross-reactivity. Positive control is GXM from H99, at concentration 100 ng/mL. Other pathogenic fungal antigens were used at concentration 10 µg/mL. Cross-reactivity was detected with *Trichosporon* sp. CYA.

The crude cytoplasmic antigens of other fungal pathogens were evaluated for mAb 18B7 ICT strip specificity. The results showed that a strong positive test was observed only with *C. neoformans* CYA. However, *Trichosporon* sp. CYA showed a slightly positive result when compared with the positive control from GXM cryptococcal capsular antigen or *C. neoformans* CYA (Figure 7).

To investigate the degree of cross-reactivity of *Trichosporon* sp. CYA, the LOD of antigen from *Trichosporon* sp. CYA was evaluated. The *Trichosporon* sp. CYA was diluted in two-fold dilution 0.125, 0.25, 0.5 and 1 µg/mL in running buffer. The LOD of *Trichosporon* sp. CYA was 0.5 µg/mL, which was higher than the LOD of cryptococcal capsular antigen by approximately 800 times (Supplementary Figure S1). 

### 3.4. Evaluation of mAb 18B7 ICT Strip with Clinical Sample and Diagnostic Performance

The diagnostic performance of the mAb 18B7 based sandwich ICT strip was further conducted using 150 clinical CSF and serum samples obtained from patients with confirmed cryptococcosis or other specified infections as well as sera from normal healthy individuals. All of the clinical samples were also tested using standard commercial kits to detect the cryptococcal capsular antigens (latex agglutination; CALAS or LFA; CrAg IMMY), four CSF samples were also positive according to India ink preparation. The negative samples from CALAS or CrAg IMMY were also collected and classified into the control group.

The total numbers of CSF samples were 53 with 28 confirmed positive and 25 confirmed negatives for cryptococcosis. Of the 28 confirmed cryptococcosis cases, 26 samples produced positive and two samples were false negatives. Of the control cases, all 25 of non-cryptococcosis samples were negative with the mAb 18B7 ICT strip. Therefore, the sensitivity and specificity of the CSF diagnosis were 92.86% and 100% respectively. The positive predictive value and negative predictive value were 100% and 93.59%, respectively, while the accuracy of the test was 96.23%. The Cohen’s kappa coefficient analysis was 0.925 for the samples analyzed by the two different diagnostic techniques, indicating ‘almost perfect agreement’ between the novel mAb 18B7 ICT strip and the standard commercial kits for diagnosis to cryptococcosis (Table 3A,B). 

For the diagnostic performance in clinical serum, the total samples were 97 including 52 confirmed cryptococcosis and 24 confirmed non-cryptococcosis. The sera of 21 normal healthy individuals were also investigated by the mAb 18B7 ICT strip, and were all negative. However, two false-negative from cryptococcosis and one false-positive from non-cryptococcosis were identified by mAb 18B7 ICT strip. So, the mAb 18B7 ICT strip exhibited a diagnostic sensitivity, specificity and accuracy of 96.15%, 97.78% and 96.91%, respectively. The positive predictive values and negative predictive values were 98.04% and 95.65%, respectively. The Cohen’s kappa coefficient analysis was 0.938 indicating ‘almost perfect agreement’ of the novel developed assay with the standard commercial assay (Table 4A,B).

## 4. Discussion

*C. neoformans* is an invasive pathogenic fungus that can cause cryptococcosis in immunocompromised hosts, especially in patients with advanced HIV/AIDs. The gold standard for cryptococcosis detection is fungal culture. However, culture has a low sensitivity and cultures must be incubated for days to weeks in order to detect the fungus. Therefore, a simple and rapid test for cryptococcal antigen assays has long been desired. Currently, the Cryptococcal Antigen Latex Agglutination System (CALAS) and Cryptococcal Antigen Lateral Flow Assay (CrAg, LFA) were globally commercialized to address this urgent need. Both commercial kits are superior in the context of diagnostic performances and are recommended by the WHO [12] for the diagnosis, prevention, and management of cryptococcosis in HIV patients. However, these kits are too costly for routine use in resource-limited countries.

The aim of this study was to develop a cost-effective in-house rapid immunochromatographic test to detect cryptococcal polysaccharide capsular antigens. We first evaluated binding of *C. neoformans* GXM with the mAb 18B7 by inhibition ELISA. mAb 18B7 recognized the capsular antigens of different serotypes of *C. neoformans* and only displayed cross-reactivity with a crude cytoplasmic antigen from *Trichosporon* sp., which produces a surface polysaccharide with the antigenic similarity cryptococcal GXM [27,28]. These results supported the further development of an ICT based on mAb 18B7.

The format of the present novel mAb18B7 ICT involved the use of a single mAb, mAb 18B7. We pursued this plan based on the idea that each unit of the GXM is expected to contain multiple repeated epitopes [29] capable of specific binding to mAb18B7. Under these conditions, the mobile phase antibody (colloidal gold conjugated mAb 18B7) binds to one of the GXM epitopes while the stationary phase antibody (immobilized mAb18B7 on test line) binds to other remaining unoccupied epitopes. Other examples of ICT developed using only a single antibody are the diagnostic tests for *Leptospira* lipopolysaccharide antigens [30] and for *Campylobacter* antigens [31]. Both of these ICTs demonstrated high degrees of diagnostic performance.

The reference commercial kit CrAg LFA was constructed with two types of anti-GXM polysaccharide antigen mAbs (mAb F12D2 and mAb 339), which were reactive with all four cryptococcal serotypes [32,33]. Prior work with mAb 18B7 showed decreasing reactivity of the mAb to different serotypes with A > B > C > D [34]. However, the reactivity against each serotype did not affect the performance of our mAb 18B7 ICT since the LOD between strains or serotypes was either similar or differed by only one dilution. The LOD of the mAb 18B7 ICT against strain H99 serotype A was 0.63 ng/mL, and other strains were also 0.63 ng/mL as well in serotypes A (strain 8710), B or D. By comparison with CrAg LFA, the LOD of mAb 18B7 ICT against cryptococcal capsule antigen was similar, with the LOD of CrAg LFA at 1 ng/mL for serotypes A and B, and 4–16 ng/mL for serotypes C and D [35]. Thus, both mAb 18B7 ICT and CrAg LFA showed high degrees of sensitivity towards different serotypes of *Cryptococcus* sp.

The mAb 18B7 ICT did cross-react with *Trichosporon* sp.; however, the LOD for antigen of *Trichosporon* sp. was significantly higher at 0.5 µg/mL (500 ng/mL, 800 times more than for cryptococcal GXM). Similarly, both standard commercial kits, CALAS and CrAg LFA cross-react with samples from disseminated trichosporonosis [11]. Previous studies by the Global Antifungal Surveillance Program (ARTEMIS) revealed that *Trichosporon* yeast was implicated as the third most commonly isolated non-candidal yeast from clinical specimens (10.7% of 8821 isolates) [36,37]; therefore, this should be considered before concluding that a positive test is indicative of cryptococcosis. In a similar way, disseminated infection due to other pathogenic basidiomycete fungi, such as *Rhodotorula* sp., should be carefully considered [11]. 

When mAb 18B7 ICT was evaluated with clinical CSF samples, the results revealed a 92.86% sensitivity and 100% specificity, when compared to the standard CrAg LFA. All 26 positive samples of mAb 18B7 ICT had CALAS titers of ≥1:10. In contrast, two samples of CSF with CALAS titers of ≤1:10 were false negatives. Moreover, all of the CSF from fungal culture positive (3 samples) and India ink positives (4 samples) were also positive with the mAb 18B7 ICT. A study with the CrAg LFA that included 832 CSF samples from individuals with HIV in South Africa and Uganda demonstrated a sensitivity of 99.3% and specificity of 99.1% [38].

For the clinical serum samples, the mAb 18B7 ICT gave a sensitivity and specificity with 97 samples of 96.15% and 97.78%, respectively. Both diagnostic criteria were lower when compared with CrAg LFA. A study of 693 sera with CrAg LFA was found to give sensitivity of 100% and specificity of 99% [39]. The two false negatives with our serum specimens were previously also negative by CALAS latex agglutination. The results of the standard kit of CrAg LFA were different from those of mAb 18B7 ICT and CALAS as reference diagnosis. Indeed, the LOD of both standard diagnostic kits against cryptococcal capsule antigens were slightly different (LOD of CrAg LFA was 1 ng/mL while LOD of CALAS was 5 ng/mL against cryptococcal capsular antigen of serotype A). There were two serum positives that required 1:4 dilutions to make the solution less viscous in order to allow the mobile phase to move to the adsorbent pad and enable interpretation of the result. One false-positive of serum occurred compared with the CrAg LFA test, which came from a patient with viral pneumonia who subsequently also grew *Aspergillus fumigatus* and *Aspergillus flavus* from bronchoalveolar lavage fluid. However, the mAb 18B7 ICT and indirect ELISA showed no cross-reactivity to *A. fumigatus* cytoplasmic mold antigen (Figure 4 and Figure 7). Future studies with more clinical samples from patients with aspergillosis and other mycoses are needed for further evaluation to better validate our mAb 18B7 ICT.

The mAb 18B7 ICT was stable at 25 °C for at least 3 months with a constant LOD (data not shown). However, the stability test reported here has only been carried out for this duration and further study with a longer period and/or an accelerated stability test is needed. Paper-based immunoassays can be compromised by high temperature and humidity, which could occur during transportation and storage in tropical areas and further testing under different environmental conditions is required to fully validate the stability of the mAb 18B7 ICT [40]. By our estimate, the costs for batch manufacturing of the mAb 18B7 ICT strip are 50 Thai baht or 1.5 $US/strip.

In summary, we describe the development of a reliable, reproducible and cost-effective rapid point-of-care diagnostic test for cryptococcosis. The mAb18B7 ICT was specifically developed for deployment in countries where commercial diagnostic kits are not available or affordable. 

## Figures and Tables

**Table 1:** Non-Cryptococcus fungal isolates studied.

Fungal Species	Isolate Number
*Talaromyces (Penicillium) marneffei*	ATCC 200051 ^δ^
*Sporothrix schenckii*	52-S1 ^†^
*Candida albicans*	ATCC 900028 ^δ^
*Candida krusei*	CI
*Candida glabrata*	CI
*Aspergillus fumigatus*	55-A1 ^†^
*Pseudallescheria boydii*	MMC60S211 ^Ϩ^
*Trichosporon* sp.	CI
*Geotrichum* sp.	CI

^δ^ Isolate from the American Type Culture Collection, Rockville, MD, USA. ^†^ Isolates from the Institute of Dermatology, Department of Medical Services, Ministry of Public Health, Bangkok, Thailand. ^Ϩ^ Isolate from culture collection in Mycology Unit, Department of Microbiology, Faculty of Medicine, Chiang Mai University, Chiang Mai, Thailand. CI: Clinical isolates from blood samples of infected patients.

**Table 2:** Clinical samples from patients with or without cryptococcosis.

Type of Clinical Samples	Case/Control Samples	Total
Cerebrospinal fluid (CSF)	Cryptococcosis -Microscopy (India ink) confirmed: 4 cases -Culture proven confirmed: 3 cases	28
	Non-Cryptococcosis ^#^	25
	**Total**	**53**
Serum	Cryptococcosis	52
	Non-Cryptococcosis ^#^	24
	Normal Healthy	21
	**Total**	**97**
**Total**		**150**

^#^ Identification lists in the Supplementary Tables S1 and S2.

**Table 3:** (**A**) Two by Two table for diagnostic performance of mAb 18B7 ICT strip against CSF samples compared with the rapid test of CrAg^®^ IMMY; (**B**) Summary of diagnostic performance of mAb 18B7 ICT strip with CSF samples.

(**A**)			
**mAb 18B7 ICT Strip**	**Rapid Test of CrAg^®^ IMMY**		**Total**
	**Cryptococcosis** **(Positive)**	**Non-Cryptococcosis** **(Negative)**	
**Positive**	26 (TP) *	0 (FP) *	**26**
**Negative**	(FN) *	25 (TN) *	**27**
**Total**	**28**	**25**	**53**
(**B**)			
**Diagnostic Performance Criteria for CSF**		**Percentages (95% C.I.)**	
Sensitivity		92.86% (76.27–99.10)	
Specificity		100% (84.24–100)	
Positive predictive value		100% (84.76–100)	
Negative predictive value		92.59% (75.53–99.04)	
Accuracy		96.23% (86.51–99.69)	
Cohen’s kappa coefficient (κ)		0.925 (0.822–1.000)	

* TP, True-Positive; TN, True-Negative; FP, False-Positive; FN, False-Negative.

**Table 4:** (**A**) Two by Two table for diagnostic performance of mAb 18B7 ICT strip against serum samples compared with the rapid test of CrAg^®^ IMMY; (**B**) Summary of diagnostic performance of mAb 18B7 ICT strip with serum samples.

(**A**)			
**mAb 18B7 ICT Strip**	**Rapid Test of CrAg^®^ IMMY**		**Total**
	**Cryptococcosis** **(Positive)**	**Non-Cryptococcosis** **(Negative)**	
**Positive**	50 (TP) *	1 (FP) *	**51**
**Negative**	2 (FN) *	44 (TN) *	**46**
**Total**	**52**	**45**	**97**
(**B**)			
**Diagnostic Performance Criteria for Serum**		**Percentages (95% C.I.)**	
Sensitivity		96.15% (86.28–99.68)	
Specificity		97.78% (87.37–99.94)	
Positive predictive value		98.04% (88.73–99.95)	
Negative predictive value		96.65% (85.16–99.47)	
Accuracy		96.91% (91.23–99.36)	
Cohen’s kappa coefficient (κ)		0.938 (0.869–1.000)	

* TP, True-Positive; TN, True-Negative; FP, False-Positive; FN, False-Negative.

## Data Availability

Data is available upon request from the authors.

## Supplementary Materials

The following are available online at https://www.mdpi.com/article/10.3390/diagnostics11050758/s1, Table S1. Clinical CSF samples from patients without cryptococcosis (control group), Table S2. Clinical serum samples from patients without cryptococcosis (control group), Figure S1. Cross-reactivity of *Trichosporon* sp. CYA was examined with the mAb 18B7 strip using concentrations of CYA from 1–0.125 µg/mL. The LOD of *Trichosporon* sp. CYA by naked eye observation was 0.5 µg/mL (red circle). (Abbreviations: CYA, Cytoplasmic Yeast Antigen; C, control line; T, test line).
