# Supplementary material for: A Novel, Inexpensive In-House Immunochromatographic Strip Test for Cryptococcosis Based on the Cryptococcal Glucuronoxylomannan Specific Monoclonal Antibody 18B7
Source: Diagnostics (Basel). 2021 Apr 23;11(5):758. doi: 10.3390/diagnostics11050758 (PMC8145812; doi:10.3390/diagnostics11050758)
Supplement: Supplementary file 1 [file diagnostics-11-00758-s001.zip › diagnostics-1193332-supplementary.pdf]

Supplementary data

Supplementary Table 1. Clinical CSF samples from patients without cryptococcosis (control group).

CSF samples (non-cryptococcosis)	Number
Horner's syndrome	1
Non-Hodgkin's lymphoma infected with *Escherichia coli* and hepatitis B	1
Systemic lupus erythematosus	3
Chronic hepatitis B	2
Neurosyphilis	2
Cellulitis	1
SLE with hepatitis C	1
Unspecified viral meningitis	1
Bacterial meningitis (*Streptococcus suis*)	1
Penicilliosis (Talaromycosis)	2
Meningitis mycoses (unspecified)	1
Bacterial meningitis (unspecified)	3
Bacterial septicemia (unspecified)	2
Tuberculosis	1
Dengue virus	1
HIV with unspecific infection	2
**Total**	**25**

Supplementary Table 2. Clinical serum samples from patients without cryptococcosis (control group).

Serum samples (non- cryptococcosis)	Number
Salmonellosis	2
Tuberculosis	1
Tuberculous meningitis	1
Non - tuberculous mycobacteria infection	1
Rheumatoid arthritis	1
Candidemia (*C. albicans*)	2
Syphilis	2
Penicilliosis (Talaromycosis)	4
Idiopathic gout	1
Viral pneumonia with *Aspergillus fumigatus* and *Aspergillus flavus*	1
Chronic hepatitis C	1
Pseudomonas aeruginosa	1
Coagulase negative staphylococci	1
Mixed infection with *Enterococcus faecium, Staphylococcus epidermidis,* Coagulase-negative staphylococci	1
Meningococcal disease	1
Bacillus cereus	1
Cysticercosis (*Taenia sp.*)	1
Malignant Neoplasm	1
**Total**	**24**

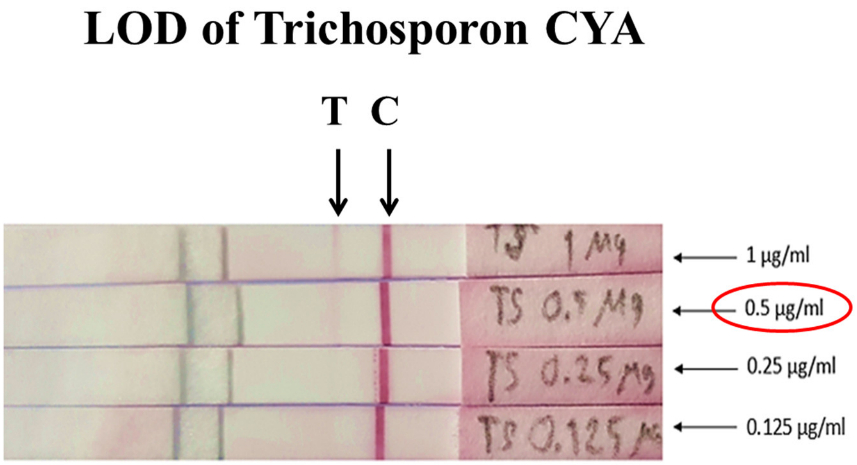

Supplementary Figure 1. Cross reactivity of *Trichosporon sp.* CYA was examined with the MAb 18B7 strip using concentrations of CYA from 1 - 0.125 µg/ml. The LOD of *Trichosporon* CYA by naked eye observation was 0.5 µg/ml (red circle). (Abbreviations: CYA, Cytoplasmic Yeast Antigen; C, control line; T, test line).
